# Immunomodulatory Effects of Mesenchymal Stem Cell-Derived Extracellular Vesicles in Allergic Airway Disease

**DOI:** 10.3390/life12121994

**Published:** 2022-11-29

**Authors:** Sung-Dong Kim, Kyu-Sup Cho

**Affiliations:** Department of Otorhinolaryngology and Biomedical Research Institute, Pusan National University School of Medicine, Pusan National University Hospital, 179 Gudeok-Ro, Seo-gu, Busan 602-739, Republic of Korea

**Keywords:** mesenchymal stem cells, extracellular vesicles, asthma, allergic rhinitis, immunosuppression

## Abstract

Mesenchymal stem cells (MSCs) have been reported as promising candidates for the treatment of various diseases, especially allergic diseases, as they have the capacity to differentiate into various cells. However, MSCs itself have several limitations such as creating a risk of aneuploidy, difficulty in handling them, immune rejection, and tumorigenicity, so interest in the extracellular vesicles (EVs) released from MSCs are increasing, and many studies have been reported. Previous studies have shown that extracellular vesicles (EVs) produced by MSCs are as effective as the MSCs themselves in suppression of allergic airway inflammation through the suppression of Th2 cytokine production and the induction of regulatory T cells (Treg) expansion. EVs are one of the substances secreted by paracrine induction from MSCs, and because it exerts its effect by delivering contents such as mRNA, microRNA, and proteins to the receptor cell, it can reduce the problems or risks related to stem cell therapy. This article reviews the immunomodulatory properties of MSCs-derived EVs and their therapeutic implications for allergic airway disease.

## 1. Introduction

Allergic rhinitis and asthma are representative atopic diseases, and 30% of allergic rhinitis patients have asthma and 70% of asthma patients have allergic rhinitis [1,2]. Allergic rhinitis and asthma are caused by various antigens and specific immunoglobulin E (Ig E), and they are immunologically characterized by the excessive activation of type 2 helper T (Th2) cells [3]. Th2 cell-induced inflammation is characterized by a significant increase in interleukin (IL)-4, IL-5, and IL-13 levels, which induce and high serum Ig E levels and airway eosinophilic inflammation and airway hyper responsiveness (AHR) [3]. The Th1 and Th2 cytokines are mutually antagonistic, and the selective suppression of Th2 responses may be crucial for protection against allergic inflammation [1,3]. There is mounting evidence that the insufficient suppression of Tregs as well as the imbalance of Th1/Th2 responses in the pathogenesis of allergic airway disease play an important role in excessive Th2 responses [4].

Stem cells are undifferentiated cells that have the potential to develop into many different cell types. They have been reported to possess the ability to self-renew in an undifferentiated state and to differentiate into many types of cells with specific functions upon receiving appropriate triggers. Stem cells can be divided into two groups, embryonic and adult. Embryonic stem cells are derived during early development at the blastocyst stage, and they are pluripotent, implying they can develop into any cell type. However, adult stem cells are multipotent, meaning they can only differentiate into certain types of tissue [5]. Both embryonic and adult stem cells have been studied as a promising source for therapeutic applications in the repair of damaged tissues and regenerative medicine [6,7,8,9].

Mesenchymal stem cells (MSCs) represent an important stem cell population with multi-potent capabilities, which means that they may have high utility for translational clinical applications. MSCs can be isolated from a number of adult tissues, and they can differentiate into several mesenchymal lineages both in vitro and in vivo, such as adipose tissue, cartilage, bone, muscle, and nerve [10,11,12,13,14].

In addition to their multi-lineage potential, MSCs have been shown to possess the unique ability to suppress an immune response and modulate inflammation. MSCs can inhibit natural killer cell functions, modulate dendritic cell maturation, and suppress the allogeneic T cell response by alternating the cytokine secretion profile of dendritic cells and T cells induced by an allogeneic immune reaction [15,16]. Therefore, MSCs have been reported to have anti-inflammatory and immunomodulatory effects in various chronic inflammatory diseases such as autoimmune encephalomyelitis, graft-versus-host disease, inflammatory bowel disease, and type I diabetes [17,18,19,20,21,22,23].

Several studies have shown that MSCs derived from adipose tissue (ASCs) and other MSCs can improve allergic airway inflammation in allergic rhinitis and asthma [24,25,26,27,28,29,30,31,32]. The immunomodulatory mechanism of MSC is related to the increase of soluble factors such as transforming growth factor-β (TGF-β) and prostaglandin E2 (PGE2) and the upregulation of Treg, but further studies are needed [24,25,26]. Furthermore, MSCs could modulate the antigen recognition of antigen presenting cells that mediate the cellular immune response, including dendritic cells, macrophages, and B cells [29,30]. However, the treatment using MSCs has some clear limitations including tumorigenicity, immune rejection, difficulty in handling them, and a risk of aneuploidy [31].

MSCs secrete a variety of autocrine and paracrine factors including angiogenic factors, chemokines, cytokines, and growth factors that have similar therapeutic effects as systemically administered MSCs do [32]. Previous several studies demonstrated that the intranasal administration of secretome, also known as a conditioned medium, is as secreted molecule from cultured MSCs can convey the same therapeutic actions as the systemic administration of the MSCs themselves [32,33,34]. The MSCs secretome can decrease an allergic airway inflammation by inhibiting Th2 cytokine release and the induction of Treg expansion even without MSCs [32,33,34]. These findings indicate that the main beneficial effects of MSCs are mediated via paracrine actions, although potentially triggered by cell-to-cell contact events. Furthermore, compared with MSCs transplantation, cell-free therapies mediated by the MSC secretome have a lot of advantages including the low possibility of immune rejection, the ease of storage, the ease of handling, and no risk of vascular occlusion or aneuploidy [35]. All of these paracrine factors can be found within the extracellular vesicles (EVs), which are cell-secreted vesicles, they are a key part of the secretome which is receiving lots of attention. Therefore, the main focus of this article is to review the immunomodulatory effects of MSC-derived EVs and to summarize the latest knowledge on clinical applications in allergic airway diseases.

## 2. Characteristics of EVs

The EV isa spherical bi-layered proteolipid secreted from the cell into the extracellular space, and it is derived by various cells such as hematopoietic cells, endothelial cells, epithelial cells, and cancer cells. The EVs are classified into 30–100 nm exosomes, 100–1000 nm microvesicles, and 1–5 μm apoptotic bodies, according to their origin, biogenesis, shape, composition, and size [36].

Exosomes, which are referred to as the intraluminal vesicles, are heterogenous in the 40–200 nm size range, and they are generated through the integration of the endosomal membrane of multivesicular bodies (MVB) and subsequent exocytosis. Additionally, exosomes are secreted by all cell types such as saliva, urine, plasma, semen, cerebrospinal fluid (CSF), bronchial fluid, synovial fluid, gastric acid, breast milk, serum, amniotic fluid, tears, lymph, and bile [37,38,39,40,41,42,43,44,45,46,47,48]. In particular, they are involved in protein storage, transportation, release, and recycling [49]. The MVB is eventually sent to the lysosome where it is either degraded along with all of its components or fuses with the cell’s plasma membrane to release its contents, including exosomes, into the extracellular space [50]. The MVB and exosome formation and release are regulated through endosomal sorting complexes that are essential for transport (ESCRT) pathways [51].

Because exosome formation and MVB transport are regulated by ESCRT proteins, these proteins are expected to be found in exosomes, and regardless of the cell type from which they originate, these proteins are termed the exosome marker proteins [52]. Although some studies indicate that there is an ESCRT-independent mechanism as another mechanism by which cells release exosomes into the extracellular space, in both of the mechanisms, CD9, CD63, and CD81 are identified as exosome-specific markers [53].

Exosomes were originally known to remove unnecessary or unwanted substances, but they have been reported to be involved in cell-to-cell communication, cell maintenance, and tumors. Several studies have reported that exosomes act as antigen-presenting vesicles to stimulate immune responses, and in the nervous system, exosomes play an important role in tissue repair and regeneration by promoting myelin formation, neurite outgrowth, and neuronal survival [54,55]. In addition, pathogenic proteins such as alpha-synuclein, superoxide dismutase, and beta-amyloid peptide which may aid in disease progression have been found to be exosomes from the central nervous system (CNS) [56,57]. The exact mechanism is not yet fully understood, and it appears that the formation of MVB can be stimulated by growth factors, and the cells modulate exosome production as needed [58].

In clinical applications and uses of exosomes, it is important to study their ability to act as carriers of biomarkers for several diseases. The exosomes in cerebrospinal fluid were found to contain alpha-synuclein, a protein related to Parkinson’s disease [59,60]. In addition, the exosomes isolated from urine were reported to reflect acute kidney injury [61], and the markers for pancreatic cancer and lung cancer were also found in exosomes [62,63]. Because exosomes are found in blood or urine, the EVs can be used to monitor the disease state of a patient or their state after the treatment of a specific disease, and changes in the biomarkers according to the course of the disease can also be evaluated [64]. It has also been reported that it can be used for immunological purposes or vaccine development because it acts as an antigen-presenting vesicle [54,65].

In addition, the exosomes can cross cell membranes and target specific cell types, making them suitable candidates for immunological applications, as well as creating drug delivery systems that move RNA and proteins to specific parts of the body through exosomes [66,67]. There are also several reports that the exosomes themselves can act as therapeutic substances to reduce tissue damage, and the potential applications and uses of exosomes have a broad range as biomarkers, drug delivery devices, and therapeutic tools in the clinical setting [68,69,70].

On the other hand, microvesicles (MVs), unlike exosomes, are relatively heterogeneous in their size, ranging from 50 to 1000 nm, and they are released either directly from the plasma membrane or through budding [71,72]. Specific protein markers that distinguish different types of EVs, MVs, exosomes, and apoptosis have not been identified, but they differ in their protein profiles due to them having different pathways of formation [73,74]. Although the proteomic profile of MVs is dependent on the method of isolation, there is a category of proteins called “marker proteins” that are proteins found in MVs irrespective of their cellular origin as a result of the biogenesis processes [75]. They are known to be clustered on the surface of plasma membrane, mainly as cytoplasmic and plasma membrane-related proteins such as tetraspanin [76]. However, specific markers for differentiating MVs from exosomes are still lacking. MVs, like exosomes, are known to be involved in intercellular communication between adjacent cells and distant cells, such as hormones, growth factors, and cytokines [77].

The apoptotic body is released into the extracellular space by the dying cell, and after the cell contracts, the hydrostatic pressure increases, and the plasma membrane of the cell is separated from the cytoskeleton, and it is formed [78]. The composition of the apoptotic bodies is in contrast to the MVs and exosomes, with there being distinct differences in their proteomic profiles [79].

As mentioned above, the released EVs contain various bioactive transmitters such as proteins, microRNAs, and mRNAs, and they play an important role in cell-to-cell communication and interaction with the surrounding microenvironment [80,81,82]. As the paracrine effect of the MSC secretome released by the MSCs which migrates into the diseased site has been reported to be an important factor in the mechanism of MSCs transplantation, the importance of stem cell-derived EVs is further emphasized in the field of stem cell application.

Gan et al. reported that EVs mediate the pathological communication between dysfunctional adipose tissue and the heart, exacerbating ischemic heart damage in obesity and diabetes. The EVs can be used as a therapeutic tool to prevent ischemic heart damage in obesity and diabetes by inhibiting the production of exosomes from abnormal adipocytes [83]. Certain regulatory microRNAs have been reported to have an immunomodulatory effect even in allergic airway disease. miR-21-5p, miR-142-3p, miR-223-3p, and miR-126-3p induce the maturation of dendritic cells, and miR 1470 modulates the immune response in asthmatics by differentiating CD4+ T cells into Treg cells via P27KIP1 [84,85,86]. In particular, miR-126-3p induces an increase in the endothelial cell function and tight junction protein expression, decreases the VEGF-α and HMGB1 levels, and miR-146a-5p inhibits group 2 innate lymphoid cells [85,87]. In addition, the MSC-EVs-derived microRNAs with biological activity in allergic airway diseases are summarized in Table 1 [88,89,90,91,92,93,94].

The mechanism by which the EVs are secreted and moved to the target cell to deliver therapeutic effects is that the EVs are secreted and contact the plasma membrane of the target cells, and then, they are absorbed through the interaction between the EVs and plasma membrane by direct fusion or endocytosis. In most cases, it moves to the lysosome, where the proteins and lipids in the EVs are degraded to supply metabolites to the target cells [80]. Since all of the EVs are surrounded by a bi-layered lipid membrane, it is possible to target the end organ cell or microenvironment, while protecting the inner factor from degrading enzymes in the blood circulation. The EVs have a biological structure similar to that of liposomes, so they are more stable in vivo than MSCs are, and it is relatively easy to improve and modify their surface properties for their application as a drug delivery system or for enhancing their therapeutic potential [75,76]. Accumulating evidence shows that administration of MSC-derived EVs have a therapeutic efficacy that is equal to or greater than that of MSC in suppressing allergic airway inflammation [32,34].

## 3. Immunomodulatory Effects of EVs for Allergic Airway Diseases

MSCs could improve the allergic airway disease by inhibiting the proliferation and function of dendritic cells which have an immunomodulatory effect, and which are differentiated into T cells and B cells [95,96,97]. The intravenous injection of MSCs significantly reduced eosinophil infiltration in the nasal mucosa and lung tissue of allergic mouse models and improved the degree of airway hypersensitivity and allergic symptoms [24,25,26]. The MSCs administrated intravenously decreased the number of Th2 cytokines such as IL-4, IL-5, IL-13, and IL-4-positive CD4+ T cells, but they increased the number of Th1 cytokine, IFN- γ and IFN- γ positive CD4+ T cells in the bronchoalveolar lavage (BAL) fluid and lung draining lymph nodes (LLNs) in an AR and asthma mouse model. The MSCs resulted in a significant decrease in the total and ovalbumin (OVA)-specific IgE and IgG1 levels. Tregs, which is characterized by the expression of transcription factor Foxp3, was significantly increased in the LLNs of asthmatic mice after the MSCs administration. Various soluble factors, including TGF-β and PGE2, are secreted by the MSCs that have migrated to the lungs by intravenous or nasal routes of administration, leading to the expansion of Tregs. Anti-inflammatory cytokines (IL-10 and TGF-β) are secreted by Tregs, which ultimately reduce the amount of pulmonary eosinophil infiltration as well as the production of allergy-specific Th2 cytokines and Ig. [24,25,26]. Additionally, in lung histology, eosinophil infiltration and inflammatory cell deposition in the peribronchial and perivascular areas were significantly decreased in the EV group compared to that of the asthma-inducing group [98].

Several studies have reported that the MSC secretomes and MSCs-derived EVs show the same immunomodulatory effects as the stem cells themselves do in allergic airway diseases [32,99,100,101]. Previous studies have shown that bone marrow, umbilical cord, and adipose tissue-derived MSCs and their EVs have the similar immunomodulatory effects in asthmatic mice. Therefore, the efficacy of MSCs-derived EVs does not depend on the MSC source tissue. Furthermore, the systemic and intranasal administration of MSC-derived EVs showed similar immunosuppressive effects in asthmatic mice [99]. De Castro et al. reported that intravenous stem cell culture media and EVs significantly reduced the degree of airway hypersensitivity and eosinophil infiltration of the lung tissues in a mouse model of asthma in the same manner as stem cells did, and the levels of IL-4, IL-5, and IL-6 were significantly reduced. Furthermore, the intravenous administration of human adipose tissue-derived MSCs and EVs reduced the total number of inflammatory cells and the ratio of eosinophils in BAL fluid, IL-5 levels in lung tissues and CD3+ CD4+ T cells in the thymus. However, the number of eosinophils in the lung tissues, the levels of IL-4, IL-13, eotaxin, and CD3+ CD4+ T cells in the BALF, and the pulmonary function showed inconsistent results [100]. Recently, intranasally administrated ASC-derived EVs significantly reduced the degree of allergic airway inflammation and improved AHR through induction of Tregs expansion in asthmatic mice. The intranasal administration of ASC-derived EVs to asthmatic mice resulted in a remarkable reduction of eosinophils and inflammatory cells in the BAL fluid, the serum total and the OVA-specific IgE levels, and the degree of eosinophilic lung inflammation. The level of IL-4 was significantly decreased in the BAL fluid and LLNs, whereas IFN-*γ* was significantly increased in the BAL fluid. Additionally, CD4^+^IL-4^+^ T cells were markedly decreased after an ASC-derived EV treatment, whereas the CD4^+^CD25^+^Foxp3^+^ T cells and CD4+IFN-*γ*+ T cells were notably increased in the LLNs of asthmatic mice [98].

In an in vitro study, the authors isolated EVs from culture supernatants of murine ASC, which were evaluated the immunomodulatory effects of EVs on Th2-mediated inflammation which was induced by Aspergillus protease antigens in mouse lung epithelial cells and primary lung epithelial cells.

Cho et al. reported that ASC-derived EVs suppressed Th2-mediated inflammation through the upregulation of TGF-β and IL-10 and the downregulation of IL-25 and eotaxin which stimulate the release and recruitment of eosinophils to the sites of inflammation synergistically with IL-5. Furthermore, the ASC-derived EVs induced an anti-inflammatory state in Th2-mediated inflammation through polarization to the M1 and M2 macrophages and dendritic cell maturation for effector T cell induction [101].

The functional enhancement of gene analysis and the microRNA expression pattern analysis, which are methods used to identify a set of overexpressed genes or proteins, have been performed, and studies on the expression pattern and differential expression characteristics of specific genes have been reported.

Kim et al. performed DNA microarray to identify the differentially expressed genes (DEGs) related to the suppression of allergic airway inflammation by ASCs-derived EVs. After the hierarchical clustering of DEGs and after the functional and pathway analysis of the potential DEGs, a total of 249 DEGs were identified, of which 21 were upregulated in the EVs group, resulting in more than 2-fold changes compared to that which was seen in the OVA group. These results suggest that PON1, Bex2, Igfbp6, and Scgb1c1 may be involved in the immunosuppressive mechanism mechanisms of MSCs-derived EVs in allergic airway diseases [102].

Paraoxonase1 (PON1) is a calcium-dependent aryldialkylphosphatase belonging to the paraoxonase (PON) family, and it has antioxidant, anti-adhesive, anti-inflammatory, anti-thrombotic, and anti-apoptotic effects. In addition to asthma, various diseases such as diabetes, rheumatism, arthritis, psoriasis, and systemic lupus erythematosus are also associated with PON1 [103,104,105,106,107,108]. The expression and activity of PON1 in asthmatic patients were significantly lower when they were compared to those of the healthy controls [104,109,110]. Furthermore, PON1 reduced the degree of airway inflammation and airway remodeling and inhibited the lipopolysaccharide (LPS)-induced inflammatory cytokine expression and lung fibroblast proliferation in asthmatic mice, thereby having significant potential effects in allergic airway disease [111]. Bex2 is a protein-coding gene known to be involved in carcinogenesis and it is a regulator of mitochondrial apoptosis and the G1 cell cycle, particularly in breast cancer [112]. Although few reports have been reported on allergic diseases, a recent study reported that Bex2 expression was suppressed by the increased DNA methylation of IL-13 which was induced in allergic airway inflammation [113]. Igfbp6 is a family of insulin-like growth factor (IGF) binding proteins related to the growth inhibitory protein regulating the availability of IGFs. The family of proteins binding to IGFs includes Igfbp1, Igfbp2, Igfbp3, Igfbp4, and Igfbp5 in addition to Igfbp6 [114]. The biological functions of Igfbp can be classified into IGF-independent and IGF-dependent actions. In particular, Igfbp has been reported as a biomarker and a therapeutic target acting on the pathogenesis of various autoimmune diseases, and Igfbp6 was associated with fibroblast proliferation and cell growth in asthmatic patients [115,116]. Fpr1 is a group of G protein-coupled cell surface receptors that have important roles in inflammation and host defenses. Since Fpr1 are expressed across a variety of cell types and interact with structurally diverse chemotactic agents, they either accelerate or inhibit the inflammatory processes upon binding to other ligands [117]. In allergic airway disease, Fpr1 has been reported to stimulate neutrophil chemotaxis and inflammatory cytokine production by phagocytes such as dendritic cells and macrophages [118]. Scgb1c1 is a member of the secretoglobin family of secreted proteins which are found in high concentrations in body fluids of the lungs, lacrimal glands, salivary glands, prostate, uterus, and in other tissues. In the human respiratory mucosa, Scgb1c1 is upregulated by IL-4 and IL-13, and it is downregulated by IFN-γ, and it plays an important role in recognizing and clearance of pathogenic microorganisms in the lung epithelial mucosa [119,120,121]. The important pulmonary genes associated with suppression of allergic airway inflammation by MSC-derived EVs are summarized in Table 2.

**Figure 1:**
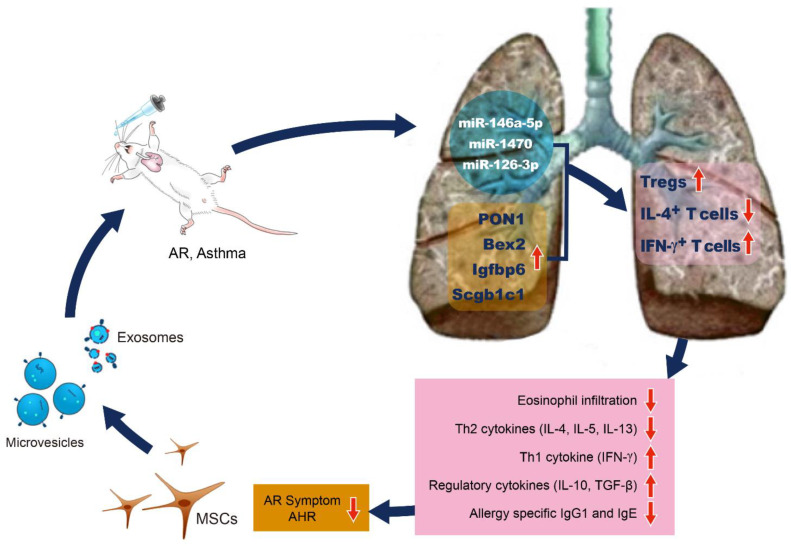
Schematic presentation of plausible mechanisms by which MSC-derived EVs regulate the allergic airway diseases. MSC-derived EVs carry microRNAs such as miR-146a-5p, miR-1470, and miR-126-3p and deliver them into lung tissues. Intranasal administration of MSC-derived EVs increases the expression of PON1, Bex2, Igfbp6, and Scgb1c1 in lung tissues of asthmatic mice. These microRNAs and pulmonary genes by MSC-derived EVs induce the expansion of Tregs. Tregs secrete IL-10 and TGF-β, which lead to decrease of allergy-specific Th2 cytokines, lung eosinophil infiltration, and allergy-specific IgG1 and IgE production.

Although studies on the mechanisms of MSCs-derived EVs on the immunomodulatory effect of ASCs are still lacking, we may present a hypothesized schematic based on previous studies. The intranasal administration of EVs isolated from the MSC secretome, including the exosomes and microvesicles, increases the expression of Bex2, PON1, Scgb1c1, and Igfbp6 in the lung tissues of asthmatic mice. These pulmonary genes induce the expansion of Tregs. Tregs secrete regulatory cytokines such as and IL-10 and TGF-β, which reduce pulmonary eosinophil infiltration, allergy-specific Th2 cytokines (IL-4, IL-5, and IL-13), allergy specific IgG1 and IgE production, allergic rhinitis symptoms, and AHR (Figure 1).

## 4. Therapeutic Implications of EVs for Allergic Airway Diseases

The ability to control the immune system in both directions is a specific advantage of EVs. To expand, the EVs have an immunomodulatory effect like dendritic cells (DCs) or regulatory T cells do, and on the other hand, they have the ability to enhance immunity like conventional vaccines do.

There are several reports of using EVs for allergic immunotherapy in allergic airway diseases. Mast cells are closely related to allergic immune responses, and they are mainly located in the intestinal or skin barrier, and they are involved in allergic sensitization and inflammation. It can also modulate the immune responses and transport the MHC molecules, which can induce the functional maturation of DCs and play important roles in the functional maturation of DCs and the efficient antigen presentation of the T cells [122]. Furthermore, it has been reported that anti-IgE treatment is the key to the treatment mechanism in allergy treatments, and mast cell-derived exosomes can neutralize IgE through the FcεRI that is displayed on the surface [123].

There is also a report using a virus-like particle-based vaccine model that can induce high protective allergen-specific IgG titers, while reducing the allergenicity of the allergen [124,125]. Several studies have reported that EVs-based vaccines can increase IgG antibody responses by inducing TH1 responses and inducing IFN-γ and TNF-α T cell responses [126,127,128].

The IgE-antigen immune complex, which is also known as IgE-promoted antigen presentation (IgE-FAP), induces CD4+ T cell and antibody responses by binding IgE and foreign antigens [129,130]. IgE-FAP contains the low-affinity receptor CD23 that is expressed on B cells, which negatively regulates the serum IgE levels by inhibiting the IgE response and downregulating the serum IgE levels [131,132,133,134]. Therefore, several studies have reported that CD23-activated B-cell derived EVs can potentially engineer IgE-promoting antigen presentation to promote T cell and antibody responses, while downregulating the serum IgE levels [135,136].

Studies using EVs as advanced therapeutics for asthma are lacking. As mentioned above, the immunomodulatory effect of the EVs have been confirmed at the pathophysiology level and in the histological findings of the asthma, but additional clinical studies are needed.

## 5. Limitations and Future Prospects of MSC-Derived EVs

Since various MSC-derived bioactive molecules are contained in MSC-derived EVs, it is necessary to secure the safety and efficacy of MSC-derived EVs by conducting additional preclinical and clinical evaluations of EVs in various disease conditions. Through the analysis of these candidate genes as well as big data-based transcriptome and proteome analyses, we may understand the common characteristics of EVs, as well as compare the unique characteristics and advantages of each of the EVs’ origins. Since the unique characteristics and advantages of each EV origin can be compared, the optimized MSC-derived EVs can be applied to an appropriate target. Nevertheless, in order to use the EVs clinically, the following limitations be considered. Firstly, since the EVs are separated from the MSCs culture medium, the culture conditions such as the number of stem cells, the volume of the medium, and the time to separate the EVs are closely related to the quantity and quality of the culture medium. For this reason, it is difficult to secure large-scale EVs derived from MSCs at high concentrations [137]. However, a recent study reported that when they were used with large capacity media, it reduces the risk of vesicle destruction and aggregation with a yield of up to 80% and high reproducibility [138].

In addition, although various methods have been introduced to isolate MSCs-derived EVs from the culture medium, their standardization and optimization are also needed [139]. In this regard, safety regulatory frameworks with good manufacturing practice standards for exosome characterization have been developed by international societies and European networks [140]. Finally, the clinical evaluation of the safety and efficacy of the MSCs-derived EVs for various diseases should be conducted, and additional studies on the precise mechanism of action are needed.

## 6. Conclusions

Many studies have been reported for the clinical application of MSCs-derived EVs in various diseases. Although the studies on MSCs-derived EVs in allergic airway diseases are still lacking in the literature, previous studies have shown that EVs have the same immunomodulatory effect as stem cells in allergic airway diseases, and they have potential as a new therapeutic agent. We believe that MSCs-derived EVs have a potential mechanism and could be novel therapeutic candidates for the treatment of allergic rhinitis and asthma in the future.

## Figures and Tables

**Table 1:** Biological activity of microRNAs associated with suppression of allergic airway inflammation by MSC-derived EVs.

microRNAs	Biological Activity
miR-146a [88]	Decreased lung inflammation; Polarization of macrophages to M2.
miR-126-3p [85]	Increased endothelial cell function; Decreased VEGF-α and HMGB1 levels; Increased tight junction protein expression.
miR-145 [89]	Increased IL-4 production and bacterial clearance; Decreased multidrug resistance-associated protein-1 expression.
miR-21-5p [90]	Decreased lung edema; M1 polarization.
miR-30b-3p [91]	Increased proliferation of alveolar epithelial cells; Decreased apoptosis of alveolar epithelial cells.
miR-1470 [86]	Anti-inflammatory properties by increasing FOXP3+ T cell.
miR-100 [92]	Decreased lung inflammation and apoptosis; Downregulation of mammalian target of rapamycin signaling.
miR-146a-5p [87]	Inhibition of group 2 innate lymphoid cells.
miR-27a-3p [93]	Decreased lung inflammation and alveolar septum thickness; Polarization of macrophages to M2 anti-inflammatory phenotype.
miR-191 [94]	Inhibition of bone marrow morphogenetic protein receptor 2.

VEGF, vascular endothelial growth factor; HMGB, high mobility group box; IL, interleukin.

**Table 2:** Pulmonary genes associated with suppression of allergic airway inflammation by MSC-derived EVs.

Genes	Description	General Characteristics	Potential Effects in Allergic Airway Disease
**PON1**	Family of PON related to calcium-dependent aryldialkylphosphatase	Antioxidant, anti-adhesive, anti-inflammatory, anti-thrombotic, and anti-apoptotic effects [103,104,105,106,107,108]	Reduced airway inflammation and airway remodeling and inhibited LPS-induced inflammatory cytokine expression and lung fibroblast proliferation in asthmatic mice [109,110,111]
**Bex2**	Family of brain expressed X-linked gene and protein-coding gene, highly expressed in brain, pancreas, and testis	Carcinogenesis, regulator of mitochondrial apoptosis and the G1 cell cycle in breast cancer [112]	Associated with inhibition of IL-13 induced in allergic airway inflammation [113]
**Igfbp6**	Family of IGFBP related to growth inhibitory protein that regulate the availability of insulin-like growth factors	Biomarker and therapeutic target acting on the pathogenesis of various autoimmune diseases [116]	Associated with fibroblast proliferation and cell growth in asthma [115]
**Fpr1**	Family of FPR, group of G protein-coupled cell surface receptors of mammalian phagocytic cells	Important roles in host defense as well as inflammatory responses including cell adhesion, directed migration, granule release, and superoxide production [117]	Associated with stimulation of neutrophil chemotaxis and inflammatory cytokine production by phagocytes such as dendritic cells and macrophages [118]
**Scgb1c1**	Family of secretoglobin secreted proteins found in high concentrations in body fluids of the lungs, lacrimal glands, salivary glands, prostate, uterus, and other tissues	Localized to Bowman’s glands in the olfactory mucosa [120]	Upregulated by IL-4, IL-13 and downregulated by IFN-γ, and it plays an important role in recognizing and clearance of pathogenic microorganisms in the lung epithelial mucosa [119,120,121]

MSC, Mesenchymal stem cells; EVs, extracellular vesicles; PON1, paraoxonase1; LPS, lipopolysaccharide; Bex2, brain expressed X-linked 2; Igfbp6, insulin-like growth factor binding protein 6; Fpr1, formyl peptide receptor 1; Scgb1c1, secretoglobin family 1C member 1; IL, interleukin; IFN-γ, interferon gamma.

## Data Availability

Not applicable.
